# Exploring the Potential Association Between Inhaled Corticosteroid and Face Aging Risk: A Mendelian Randomization Study

**DOI:** 10.3390/ph18060846

**Published:** 2025-06-05

**Authors:** Junpeng Li, Yaqiong Liu, Gujie Wu, Shanye Yin, Lin Cheng, Wenjun Deng

**Affiliations:** 1 Department of Surgery Tohoku, University Graduate School of Medicine, Sendai 980-8575, Japan; li.junpeng.t1@dc.tohoku.ac.jp; 2 Regenerative Medicine Institute (REMEDI), University of Galway, H91 TK33 Galway, Ireland; y.liu14@universityofgalway.ie (Y.L.); gujiewu08@gmail.com (G.W.); 3 Department of Pathology, Albert Einstein College of Medicine, Bronx, NY 10461, USA; shanye.yin@einsteinmed.edu; 4 Department of Neurology, Massachusetts General Hospital, Harvard Medical School, Boston, MA 02114, USA

**Keywords:** inhaled corticosteroids, face aging, drug-target-mendelian randomization, drug–side effect

## Abstract

**Background**: Asthma is one of the most prevalent chronic diseases, affecting more than 300 million individuals globally. Inhaled corticosteroids (ICSs) are recommended as the primary therapy for managing and preventing asthma symptoms in current treatment guidelines. However, long-term use of ICSs could lead to multiple side effects, including skin changes. **Methods**: We identified ICS target genes using DrugBank and DGIdb databases and derived genetic instruments from cis-eQTL data in whole-blood samples (n = 31,684). GWAS data for facial aging traits (n = 423,999) and plasma metabolites (1400 metabolites, n = 8000) were analyzed. DNA methylation QTL (mQTL) data were used to explore epigenetic regulation. Mendelian randomization (MR) and colocalization analyses were performed to assess causality and shared genetic loci. **Results**: MR analysis suggested a significant link between genetically proxied ICSs (ORMDL3) and face aging in the European population. Further mediation analysis indicated that 5-Hydroxylysine partially mediates the relationship between ICSs and face aging. In addition, our analysis revealed the pleiotropic association of some novel DNA methylation sites of ORMDL3 with face aging, suggesting the possible regulatory mechanism that are involved in face aging. **Conclusions**: These findings, while exploratory, raise the hypothesis that ICSs may impact face aging through upregulation of ORMDL3 expression and 5-hydroxylysine metabolism and highlight the need for further pharmacological and clinical research to validate these potential effects.

## 1. Introduction

Asthma is a complex condition defined by bronchoconstriction hyper-reactivity and intermittent airflow obstruction, affecting an estimated 300 million people globally [1]. The primary clinical manifestations of asthma include coughing, wheezing, and difficulty in breathing [2]. In current treatment guidelines, inhaled corticosteroids (ICSs) and short- and long-acting β2-adrenoceptor agonists are recommended for controlling asthma symptoms. However, research has suggested that long-term use of ICSs could lead to multiple side effects, such as weight gain, infectious complications, osteoporosis [3], extensive adrenal suppression, glaucoma, and cataracts [4].

ICS use has also been related to an increased risk of various types of cutaneous lesions, including, but not limited to, Cushing syndrome, atrophy, striae, and alterations in skin extensibility and elasticity [5]. Although a questionnaire-based survey revealed that skin changes were the second most commonly reported adverse effect linked to the use of ICSs [6], few randomized controlled trials have estimated the potential skin changes induced by ICSs. So far, the primary understanding of the mechanisms behind corticosteroid-induced skin changes has been largely based on studies involving topical corticosteroids. Topical corticosteroids have been found to suppress the production of collagen and hyaluronic acid by dermal fibroblasts, as well as hinder cell proliferation [7,8,9].

In the setting of limited clinical trial and experimental evidence, Mendelian randomization (MR) could provide a cost-effective solution to explore the potential effects of ICSs on skin [10]. In this study, we hypothesized that ICSs induce alterations in the DNA methylation levels of risk genes, thereby resulting in face aging. First, we used expression quantitative trait loci (eQTL) data as the genetic instruments for each target gene of ICSs. Then, we performed two-sample MR using eQTL data and a public genome-wide association study (GWAS) of face aging to identify risk genes. For these risk genes, we examined DNA methylation QTLs (mQTLs) in blood to explore potential epigenetic regulation. Furthermore, single-cell sequence (scRNA-seq) data showed the expression level of risk genes in fibroblasts. Finally, we assessed whether plasma metabolites could mediate associations between these ICS-target genes and face aging. Together, these findings may provide initial insights into the potential side effects of long-term use of ICSs on face aging in European populations.

## 2. Results

### 2.1. Causal Effects of Drug Targets on Facial Aging

**Figure 1:**
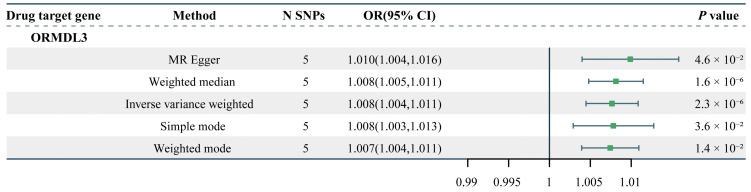
The two-sample MR result of the causal effects of inhaled corticosteroid drug target gene ORMDL3 on facial aging risk. N SNPs, number of SNPs; OR, odds ratio; CI, confidence interval.

After applying FDR correction, our Mendelian randomization analysis identified that increased expression of the ORMDL3 (orosomucoid-like 3) gene is associated with an elevated risk of facial aging (OR = 1.008; 95% CI, 1.00447–1.0108; nominal *p* = 2.3 × 10^−6^; PFDR = 3.9 × 10^−5^) (Figure 1 and Supplementary Table S1). To assess the robustness of our MR results, we conducted a heterogeneity analysis (*p* = 0.24) and pleiotropy tests (*p* = 0.44), both of which showed *p* values > 0.05, indicating that the MR results are stable and reliable.

To further validate the findings, we performed an IVW MR analysis using the GTEx V8 whole-blood dataset (Supplementary Table S2). The results demonstrated a significant association between ORMDL3 expression and facial aging risk in the GTEx dataset as well (OR = 1.0102; 95% CI, 1.0058–1.0147; *p* = 6.97 × 10^−6^). Heterogeneity analysis (*p* = 0.33) and pleiotropy tests (*p* > 0.05) further supported the robustness of the MR findings.

**Figure 2:**
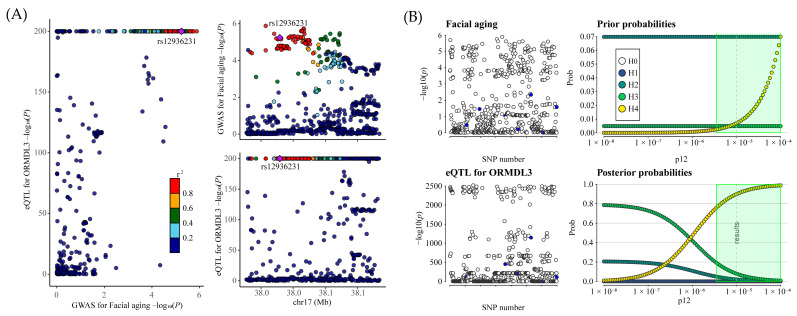
(**A**) The LocusCompare plot illustrates the colocalization between eQTL data for ORMDL3 and GWAS data for facial aging. The plots highlight the lead SNP, identified as having the highest posterior probability based on coloc analysis, with other SNPs color-coded according to their LD r2 relative to the lead SNP. (**B**) Sensitivity analysis evaluates the colocalization between ORMDL3 and facial aging under the criteria of H4 > 0.75 and H4/H3 > 3. The left panels depict local Manhattan plots for the two traits, while the right panels present prior and posterior probabilities for H0–H4 as a function of p12. The dashed vertical line marks the p12 value used in the primary analysis around which sensitivity is assessed. In the green-shaded region, the colocalization findings appear highly robust. SNP refers to single-nucleotide polymorphism.

To confirm the reliability of our Mendelian randomization findings, we additionally implemented a colocalization analysis between ORMDL3 expression in blood and facial aging. The results showed a posterior probability of 100% for shared genetic variants (rs12936231) between ORMDL3 expression and facial aging, providing strong evidence for the validity of hypothesis 4 (Figure 2A and Supplementary Table S3). Sensitivity analyses for colocalization further confirmed the robustness of the results (Figure 2B).

These findings collectively indicate a stable and significant causal relationship between increased ORMDL3 gene expression and an elevated risk of facial aging.

### 2.2. Gene Expression Analysis by Single-Cell Clusters

**Figure 3:**
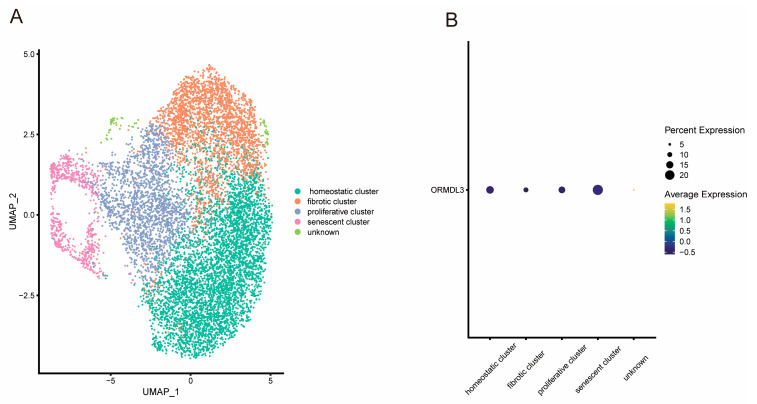
Single-cell analysis of human dermal fibroblast (ASF-4-1 cell line). (**A**) UMAP plot shows the cell clusters of dermal fibroblast; (**B**) The dot plot depicts expression levels of ORMDL3 in each fibroblast cluster.

Then, we investigated the expression level of ORMLD3 in human dermal fibroblasts (ASF-4 cell line) using scRNA-seq data. The results showed that these cells were mainly divided into four clusters: a homeostatic cluster, a proliferative cluster, a fibrotic cluster, and a senescent cluster (Figure 3A). Interestingly, we found that ORMLD3 had a higher expression level in the senescent cluster than in other clusters (Figure 3B), indicating its potential association with the senescent dermal fibroblast phenotype.

### 2.3. Causal Effect of DNA Methylation at ORMDL3-Associated CpG Sites on ORMDL3 Gene Expression

**Figure 4:**
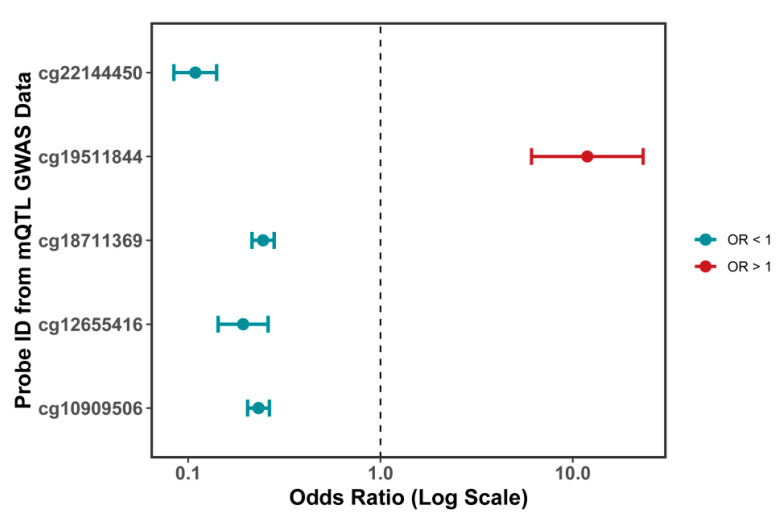
Association between predicted ORMDL3 gene methylation (mQTL) and ORMDL3 gene expression from Mendelian randomization analysis.

To investigate the causal impact of DNA methylation at CpG sites linked to ORMDL3 on the gene’s expression, we performed a two-sample Mendelian randomization (MR) analysis. This approach utilized methylation quantitative trait loci (mQTL) data in conjunction with expression quantitative trait loci (eQTL) data for ORMDL3 (refer to Supplementary Table S4). As shown in Figure 4, the methylation levels of specific CpG probes were significantly associated with ORMDL3 gene expression. Specifically, the MR analysis identified five CpG sites that were significantly associated with ORMDL3 expression, including cg22144450, cg19511844, cg18711369, cg12655416, and cg10909506.

To better illustrate the magnitude of these effects, the odds ratios (OR) were calculated on a logarithmic scale. The results showed that cg22144450, cg18711369, cg12655416, and cg10909506 had OR values less than 1, indicating that methylation at these sites negatively regulated ORMDL3 expression (cg22144450: odds ratio = 0.109, 95% confidence interval (CI): 0.084–0.141, *p*-value < 0.001; cg18711369: odds ratio = 0.245, 95% CI: 0.215–0.280, *p*-value < 0.001; cg12655416: odds ratio = 0.193, 95% CI: 0.143–0.260, *p*-value < 0.001; cg10909506: odds ratio = 0.232, 95% CI: 0.204–0.264, *p*-value < 0.001). In contrast, cg19511844 exhibited an OR greater than 1, suggesting that methylation at this site significantly promoted ORMDL3 gene expression (cg19511844: OR = 11.907, 95% CI: 6.102–23.235, *p* < 0.001).

**Figure 5:**
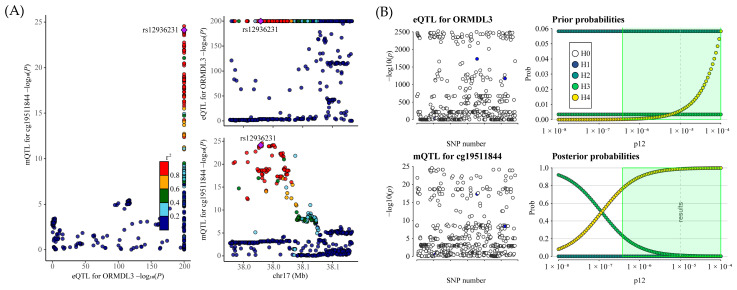
(**A**) The LocusCompare plot showing colocalization of mQTL data for cg19511844 and eQTL data for ORMDL3. We selected rs12936231 as the lead SNP to maintain consistency with previous eQTL-GWAS analyses, where it had the highest posterior probability. Other SNPs are color-coded based on their linkage disequilibrium (LD) with rs12936231, as indicated by their LD r^2^ values. (**B**). Sensitivity analysis for colocalization between cg19511844 and ORMDL3, using H4 > 0.75 and H4/H3 > 3 as the cutoff. The left-hand panels present local Manhattan plots emphasizing the specific genomic region under investigation. The right-hand panels illustrate the prior and posterior probabilities for the colocalization hypotheses H0–H4 as they vary with p12, with the dashed line indicating the p12 value applied in the initial analysis. Within the green-shaded area, the colocalization outcome appears to be consistent and reliable.

To further validate the robustness of these associations, we performed a colocalization analysis. Given that ORMDL3 is a risk gene for facial aging and that CpG methylation sites negatively affecting its expression were excluded from subsequent analysis, we focused on the colocalization evaluation of cg19511844 methylation and ORMDL3 expression (Figure 5A and Supplementary Table S5). Using rs12936231 as the primary single-nucleotide polymorphism (SNP), we observed a posterior probability of colocalization (PPH4) of 98.90% for shared causal variants between ORMDL3 expression and cg19511844 methylation. Additionally, rs12936231 exhibited a PPH4 of 100%.

Sensitivity analyses for colocalization (Figure 5B) further confirmed the robustness of these findings, providing strong evidence for a causal relationship between cg19511844 methylation and increased ORMDL3 gene expression.

### 2.4. Causal Effects of ORMDL3-Related Methylation on Facial Aging Risk

To further evaluate the direct causal effect of cg19511844 methylation on facial aging risk, a two-sample MR analysis was conducted using raw mQTL data generated from two cohorts, BSGS (n = 614) and LBC (n = 1366), in peripheral blood, along with summary statistics on facial aging risk from the IEU GWAS/UK Biobank (Supplementary Table S6).

**Figure 6:**
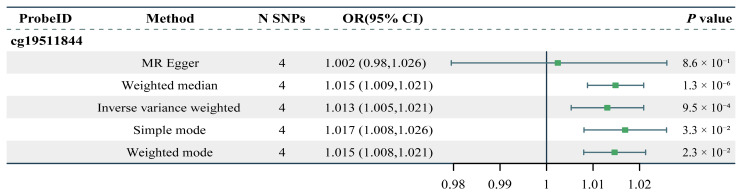
The two-sample MR result of the causal effects of methylation at cg19511844 on facial aging risk. N SNPs, number of SNPs.

Figure 6 presents the results of the MR analysis assessing the impact of cg19511844 methylation associated with ORMDL3 on facial aging risk. The findings indicate a significant positive association between cg19511844 methylation and increased facial aging risk (OR = 1.013, 95% CI: 1.005–1.021, *p* < 0.001), suggesting that elevated methylation levels at this site increase the risk of facial aging.

**Figure 7:**
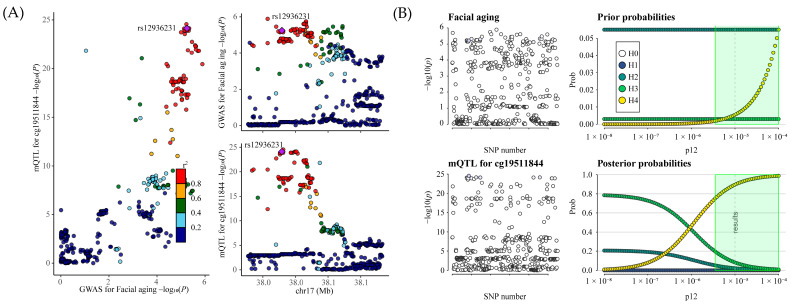
(**A**) LocusCompare plots contrasting mQTL findings for cg19511844 with GWAS outcomes related to facial aging. We selected rs12936231 as the lead SNP to maintain consistency with previous eQTL-GWAS analyses, where it had the highest posterior probability. Other SNPs are color-coded based on their linkage disequilibrium (LD) with rs12936231, as indicated by their LD r^2^ values. (**B**) Sensitivity analysis for colocalization between cg19511844 and facial aging, using criteria of H4 > 0.75 and H4/H3 > 3. The panels on the left present local Manhattan plots showcasing the genomic region of focus. Those on the right depict the prior and posterior probabilities associated with colocalization hypotheses H0–H4 as they depend on p12, with a dashed line indicating the p12 value applied in the initial analysis. Within the green-shaded area, the evidence for colocalization appears strong and consistent.

To further evaluate the robustness of the association, a colocalization analysis of cg19511844 methylation and facial aging risk was performed (Figure 7A and Supplementary Table S7). Using rs12936231 as the lead SNP, we observed a posterior probability of shared causal variants (PPH4) of 89.64%, indicating an 89.64% likelihood that the changes in methylation levels and increased facial aging risk are driven by the same genetic variant. Sensitivity analyses further confirmed the robustness of the results (Figure 7B), providing strong evidence for the causal relationship between cg19511844 methylation and increased facial aging risk.

### 2.5. Mediation Analysis

Previous studies have suggested that ICS may influence metabolite levels by altering lipid metabolism and glucose metabolism, thereby participating in the regulation of cellular function, inflammatory responses, and oxidative stress processes, ultimately accelerating aging. Thus, we employed a two-step MR analysis to evaluate whether plasma metabolites mediate the relationship between ORMDL3 gene expression and facial aging risk. A total of 1400 plasma metabolites were selected as potential mediators to analyze their causal effects.

First, we used genetic variants associated with ORMDL3 gene expression as IVs to estimate their causal effect on the levels of 1400 plasma metabolites. The analysis identified 77 metabolites that showed significant associations in the MR analysis (IVW method, *p* < 0.05). Specifically, ORMDL3 gene expression was found to significantly increase the levels of 30 metabolites (e.g., 5-hydroxylysine, OR = 1.045, *p* = 0.03), while decreasing the levels of 47 metabolites (e.g., citrulline, OR = 0.95, *p* = 0.02) (see Supplementary Tables S8 and S9).

In the second stage, metabolites identified as significantly associated in the initial step were used as exposure factors to evaluate their causal link to facial aging. The findings revealed that elevated levels of 5-hydroxylysine were strongly correlated with a higher likelihood of facial aging (odds ratio = 1.012 [95% CI: 1.005–1.018], *p*-value < 0.001) (refer to Supplementary Table S10).

To verify the directionality of the causal relationship, we conducted a reverse MR analysis using facial aging as the exposure variable. The results did not show any significant reverse causal effect (Supplementary Table S11), further supporting the unidirectional causal relationship in which ORMDL3 gene expression increases the risk of facial aging by regulating metabolite levels.

Finally, based on mediation analysis, we evaluated the role of 5-hydroxylysine as a mediator in the relationship between ORMDL3 gene expression and facial aging risk. The results showed that the indirect effect of 5-hydroxylysine (β = 0.0004959) accounted for 6.53% of the total effect of ORMDL3 gene expression on facial aging (Supplementary Table S12), indicating that 5-hydroxylysine partially mediates this biological process.

## 3. Discussion

This study employed MR analysis to explore the potential causal relationship between genetically proxied ICSs and the risk of facial aging using public eQTL and GWAS data. Our results indicated that elevated expression of ORMDL3 (a target gene of fluticasone) could contribute to an increased risk of facial aging. Mediation analysis further indicated that 5-Hydroxylysine acts as an intermediary in the relationship between ORMLD3 and face aging. The DNA methylation sites of ORMDL3 regulated its expression, thereby increasing the risk of face aging. Moreover, colocalization analysis showed that mQTLs of cg19511844 overlap with SNPs of ORMDL3 and face aging. Our study tries to fill the gaps in the understanding of the association between ICS treatment and face aging in a genomic context. However, these findings require further experimental and clinical validation.

Treatment with ICSs has been shown to enhance the expression level of the ORMDL3 gene in children with atopic asthma [11]. Our results indicated that higher ORMLD3 expression resulted in increased face aging. Studies have revealed that ORMLD3 modulates endoplasmic reticulum-mediated calcium signaling [12], leading to an unfolded protein response, which can initiate inflammatory responses [13]. In addition, ORMDL3 has been reported to participate in the regulation of sphingolipid metabolism [14]. Sphingolipids, as key components of cell membranes, are essential for various cellular functions, including cell proliferation, signal transduction, and apoptosis [15]. Nevertheless, whether these functions explain the link between ORMDL3 expression and facial aging remains uncertain. To further investigate this association, we examined the expression of ORMDL3 in human skin. The skin comprises three layers: the epidermis, dermis, and subcutaneous tissue. Dermal fibroblasts, which are located within the dermis, are crucial in the synthesis and maintenance of the extracellular matrix (ECM) components, such as collagen, elastin, and glycosaminoglycan. These cells are essential for maintaining the structural integrity and elasticity of the skin, and they play a key role in wound healing, skin regeneration, and response to external stressors. However, with aging, dermal fibroblasts may become senescent, leading to an increased production of reactive oxygen species and ECM-degrading enzymes. These factors contribute to the breakdown of collagen and other ECM components, resulting in skin weakening, fragmentation of the structural matrix, and visible signs of aging, including wrinkles and reduced elasticity [16]. Using scRNA-seq data, we found that ORMDL3 expression was significantly elevated in a senescent cluster of human dermal fibroblasts. Furthermore, previous studies suggest that ORMDL3 knockout increases sphingolipid levels in keratinocytes, which are vital for maintaining the skin’s barrier function [17]. In summary, we propose that ORMDL3 may accelerate the structural deterioration of the skin and contribute to facial aging through three potential mechanisms: (i) promoting dermal fibroblast senescence, (ii) disrupting sphingolipid metabolism and impairing skin barrier function, and (iii) activating inflammatory and cytokine signaling pathways. Despite these findings, these mechanisms remain hypothetical and require further functional experiments to elucidate how ICSs may increase the risk of facial aging through ORMDL3-mediated pathways.

Substantial evidence shows that blood metabolites are closely linked to skin aging [18]. The metabolic pathways of glucose, proteins, and lipids can have a substantial impact on skin aging. Abnormal glucose metabolism can result in the overproduction of reactive oxygen species and the accumulation of advanced glycation end products, leading to skin damage and accelerated aging [19]. Protein is a crucial indicator of both healthy and aging skin. The breakdown of collagen [16] and a reduction in glutamine levels [20] can influence the skin’s structure and functionality. Similarly, lipids play a vital role in maintaining the skin’s protective barrier, but lipid peroxidation products contribute to aging and water loss from the skin [21]. Due to the close association between blood metabolites and aging skin, we investigated whether metabolites serve as a mediator in the causal relationship between ORMDL3 and face aging. Mediation analysis revealed that 5-hydroxylysine levels mediated 6.53% of the overall effect of ORMDL3 gene expression on the risk of facial aging. Although the relationship between 5-hydroxylysine and ORMDL3 is not clear yet, current studies show that 5-hydroxylysine is typically found in collagen as a glycosylated form [22]. Collagen’s primary structure includes the repetitive Gly-X-Y sequence, where proline or lysine typically occupy the Y position. During synthesis, some of these residues are hydroxylated, promoting collagen folding into a stable triple helix structure [22]. This triple helix, maintained by the Gly-Pro-Hyp triplet, is stabilized further by prolyl-4-hydroxylase, which hydroxylates proline residues at the Y position to form hydroxyproline [22]. Hydroxylysine enhances collagen’s triple-helix stability, which is essential for the structural integrity of tissues. Disruptions in this stability can lead to diseases, as abnormal hydroxylation patterns affect collagen’s resilience and increase production rates, which can contribute to pathological tissue changes [23,24,25].

Our mQTL analysis revealed that DNA methylation at the CpG site cg19511844, located within the first intron of the ORMDL3 gene (chromosome 17q21.1), significantly increases the expression of ORMDL3, which may in turn contribute to facial aging. This CpG site resides within a potential regulatory region of the ORMDL3 gene and may enhance its transcriptional activity through influencing transcription factor binding, chromatin accessibility, or RNA polymerase recruitment [26,27]. Although cg19511844 itself has not been investigated in previous studies, several other CpG sites within the ORMDL3 gene body (such as cg12655416, cg22144450, and cg18711369) have been reported to be significantly associated with ORMDL3 expression in peripheral blood leukocytes and CD4^+^ T cells, supporting the role of local DNA methylation in regulating ORMDL3 transcription [28]. Furthermore, our colocalization analysis demonstrated substantial overlap between genetic variants associated with cg19511844 methylation (mQTLs) and those influencing ORMDL3 expression (eQTLs) and facial aging risk (GWAS signals). These findings suggest that the observed phenotypic changes may be driven by a shared set of causal variants, thereby supporting a common genetic mechanism linking increased ORMDL3 expression to elevated facial aging risk.

This study has several limitations that should be noted. First, our analysis focused only on drug targets identified in the DGIdb and DrugBank databases, without considering potential off-target effects of inhaled corticosteroids (ICSs), which may also contribute to facial aging. Second, although our study aimed to explore the genetic and epigenetic basis of ICS-associated facial aging, we relied on publicly available eQTL and mQTL data derived from blood tissues due to the scarcity of skin-specific datasets. While the use of large-scale blood data enhanced statistical power, it may not have fully captured tissue-specific regulatory mechanisms in dermal fibroblasts. In addition, the study population was limited to individuals of European ancestry, which may restrict the generalizability of the findings to other ethnic groups. Due to the lack of sex-stratified data on facial aging, we were also unable to assess potential sex-specific differences in response to ICSs. Third, the inherent limitations of MR should be considered, including the possibility of pleiotropy (i.e., a single SNP influencing multiple traits), horizontal pleiotropy, and heterogeneity of effects. Fourth, although MR reduces confounding to some extent, environmental and lifestyle factors—such as ultraviolet (UV) exposure [29], smoking, diet, air pollution, and psychological stress [30]—may still significantly impact the results and were not fully accounted for in this study. Moreover, determining the actual effects of drug exposure in real-world clinical settings may be challenging based on the effect sizes estimated from MR analysis, as exposure duration, treatment dose, individual metabolic differences, or drug-binding affinity can all influence drug efficacy [31]. Fifth, the specific mechanisms by which the target genes and associated genetic variants act on facial tissues remain unclear, and the proposed molecular pathways lack support from experimental, clinical, or strong epidemiological evidence. Lastly, our scRNA-seq data were obtained from a single human dermal fibroblast cell line (ASF-4), and the results from a single cell line may not represent the overall characteristics of fibroblast populations in vivo. Therefore, further experimental validation and studies involving more diverse populations are needed to strengthen and extend our findings.

In conclusion, our study suggested that the long-term use of ICSs may be associated with face aging, although this remains a hypothesis-generating finding that requires further clinical and experimental validation. ORMDL3 was identified as a candidate risk gene contributing to this putative association, and integrative analyses of DNA methylation and plasma metabolites provided additional insights into possible underlying mechanisms. Given the theoretical nature of these results, we recommend that clinicians remain aware of potential skin-related effects during ICS therapy, but emphasize that further pharmacokinetic, tissue-specific, and clinical studies are needed to validate these findings.

## 4. Materials and Methods

### 4.1. Study Design and Data Resources

**Figure 8:**
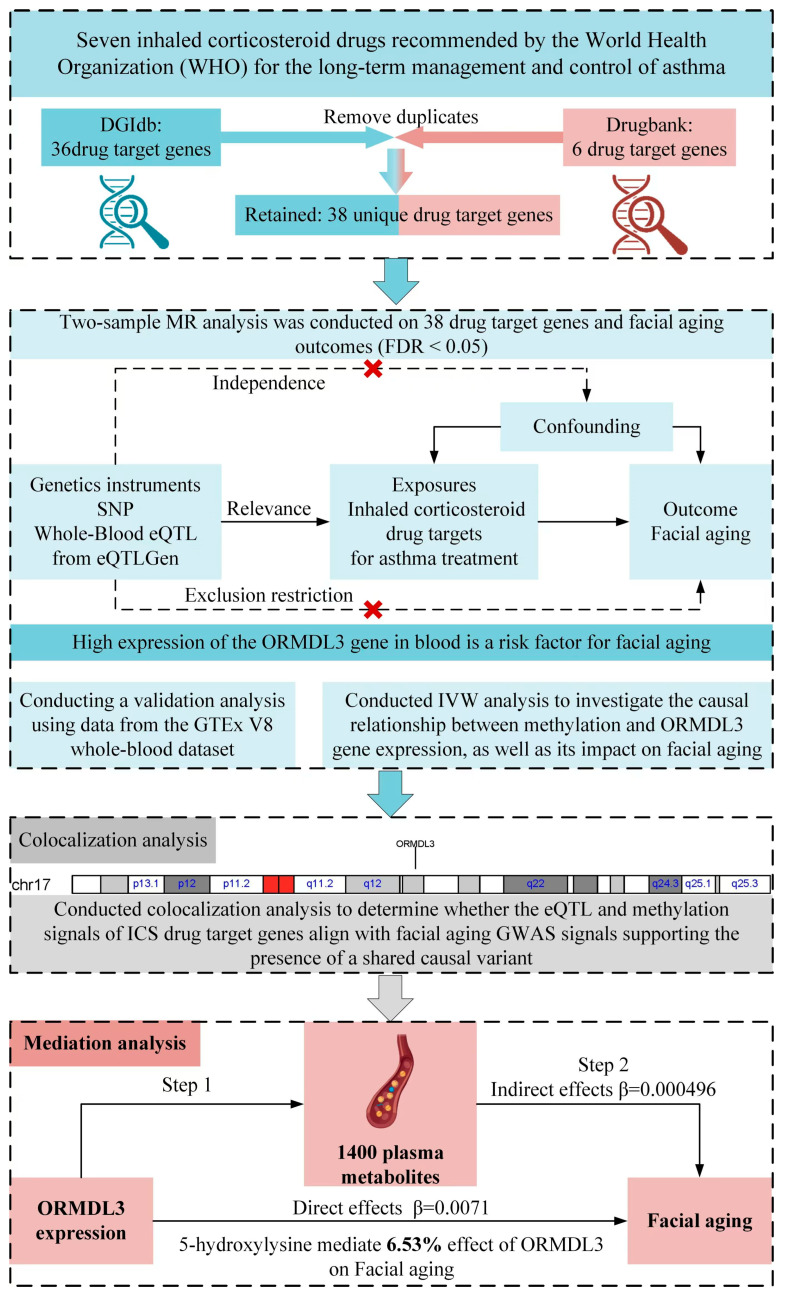
Overview of the study framework and key findings.

Figure 8 provides a visualization of the complete research framework. The GWAS data employed in this investigation were drawn from open-access sources, as listed in Supplementary Table S13. All exposure and outcome data were derived from European populations. The blood eQTL data used in the analysis were sourced from the IEU GWAS/eQTLGen project, which includes blood samples from 31,684 European participants. Additionally, eQTL data from the GTEx V8 whole-blood dataset were used for validation. This dataset predominantly includes individuals of European ancestry (~85%) and contains 670 samples. Data on facial aging were obtained from the IEU GWAS/UK Biobank project, which included 423,999 individuals of European ancestry. Moreover, mQTL data were obtained from two separate cohorts, BSGS (n = 614) and LBC (n = 1366), both of which analyzed peripheral blood samples. Plasma metabolite data were sourced from GCST90199621 to GCST90201020, comprising 8000 European participants.

As an inclusion criterion, we used large-scale public datasets derived from European populations. All datasets applied established quality control pipelines, including ancestry inference, to ensure completeness of genotype and phenotype data. Although no additional ancestry filtering was performed on the GTEx V8 dataset, it consists of approximately 85% European-ancestry individuals and was primarily used for validation purposes.

### 4.2. Identification of Target Genes for Seven Inhaled Corticosteroid Drugs

Using the DrugBank and DGIdb databases, the target genes for seven inhaled corticosteroids, endorsed by the World Health Organization (WHO) for long-term asthma management, were identified (Table 1). The interaction scores between these drugs and their target genes, along with the relevant literature, were thoroughly documented in the DGIdb database, with detailed information available in Supplementary Table S14.

### 4.3. Genetic Instruments for Target Genes of Inhaled Corticosteroid Drugs

We selected genetic instruments for the target genes of ICSs from the publicly available eQTLGen 2019 dataset, which includes both cis- and trans-eQTLs in whole blood (Supplementary Table S15). To identify suitable genetic instruments for each targeted gene, we first isolated single-nucleotide polymorphic loci (SNPs) strongly linked to alterations in gene expression. These variants were chosen only if they surpassed an exacting genome-wide statistical threshold (*p* < 5 × 10^−8^), after which a stringent linkage disequilibrium (LD) filtration step (r^2^ < 0.001) was applied, restricting the assessment to a focused genomic region of 10,000 kilobases. In subsequent analyses, we designated the highest-priority cis- and trans-acting expression quantitative trait loci (eQTLs) for each gene as the instrumental variables (IVs) employed in the Mendelian randomization framework. By implementing these refined criteria and procedures, our approach ensures that the selected genetic instruments more accurately capture the causal influence of gene expression on downstream phenotypic outcomes.

To further validate the results, we used eQTL data from the GTEx V8 whole-blood dataset. This dataset consists of samples predominantly from individuals of European ancestry (~85%) and includes 670 samples. For the validation analysis, all selected eQTLs were cis-eQTLs, chosen according to the following criteria: genome-wide significance threshold (*p* < 5 × 10^−8^), LD clumping (r^2^ < 0.2), and a window size of 250 kb. Compared with the primary dataset, a more relaxed LD clumping standard was applied to the GTEx validation dataset. This adjustment was necessary due to the smaller sample size of the GTEx dataset, where a relaxed LD parameter improves the detection rate of genetic instruments and avoids missing potential causal variants. Additionally, the cis-eQTLs’ local regulatory characteristics make broader LD thresholds more suitable for capturing local effects, thereby enhancing the sensitivity of the validation analysis (Supplementary Table S16).

We also selected mQTL associated with ORMDL3 gene expression from two cohorts, BSGS (n = 614) and LBC (n = 1366), both based on peripheral blood mQTL data (Supplementary Table S17). These genetic instruments were selected using a *p*-value threshold (*p* < 5 × 10^−5^) and were subjected to local LD clumping (r^2^ < 0.1) with a window size of 10,000 kb. These instruments were used to evaluate the potential effects of methylation on gene expression and related phenotypes.

To ensure the strength of the selected genetic instruments, we calculated R^2^ and F-statistics. The formula for R^2^ is R^2^ = 2 × EAF × (1 − EAF) × beta^2^, and the F-statistic is calculated as F = R^2^ × (N − 2)/(1 − R^2^), where EAF represents the effect allele frequency, beta is the effect size, and N is the sample size. R^2^ measures the variance in gene expression explained by the SNP, while the F-statistic assesses the strength of the genetic instruments. Higher F-statistics indicate stronger instruments, minimizing bias caused by weak instruments. The combination of cis- and trans-QTLs broadens the scope of our analysis, capturing both local and distal regulatory effects on gene expression. This comprehensive approach ensures that the selected genetic instruments reliably evaluate the effects of ICS drug target genes on facial aging and the potential effects of methylation on gene expression and facial aging in the MR analysis.

We selected eQTLs associated with ICS target gene expression as instrumental variables based on statistical thresholds (*p* < 5 × 10^−8^, r^2^ < 0.001, F-statistic > 10). Target genes were identified using known drug–gene interactions from the DrugBank and DGIdb databases. eQTLs were used as proxies for the long-term effects of ICS exposure. MR–Egger regression and Cochran’s Q test were used to assess and exclude significant pleiotropy and heterogeneity.

### 4.4. Mendelian Randomization Analysis

In this study, we primarily used the IVW meta-analysis method as the main approach to assess causal relationships. IVW operates under the assumption of no horizontal pleiotropy, estimating the overall impact of gene expression on facial aging and the influence of methylation on gene expression and facial aging by weighting the effect of each SNP. To detect and adjust for pleiotropy, we employed MR–Egger regression, using the Egger intercept to assess pleiotropic bias. A significantly non-zero Egger intercept indicates the presence of horizontal pleiotropy. The pleiotropy *p*-value was used to evaluate the significance of pleiotropic effects, with a value less than 0.05 suggesting that certain SNPs may exhibit significant pleiotropy. Cochran’s Q test was used to evaluate the heterogeneity of SNP effects, with the Q statistic and corresponding *p*-value (heterogeneity) indicating the presence of significant heterogeneity. When significant heterogeneity was detected by the Q test, a random-effects IVW model was applied to better capture variations in SNP effects. To control for false positives due to multiple comparisons, Benjamini–Hochberg false discovery rate (FDR) correction was applied to adjust all *p*-values, ensuring the statistical robustness of significant results. The threshold for statistical significance after correction was set at PFDR < 0.05. All Mendelian randomization results are reported as odds ratios (ORs) with 95% confidence intervals (CIs), quantifying the associations between gene expression and facial aging risk, as well as methylation, gene expression, and facial aging risk.

### 4.5. Colocalization Analysis

Based on MR evidence (FDR < 0.05), we further conducted colocalization analyses to verify whether the eQTL signals of ICS drug target genes colocalize with facial aging GWAS signals, and whether the ORMDL3 methylation signals colocalize with ORMDL3 gene expression and facial aging GWAS signals at the same genetic loci. By assessing the overlap among gene expression, methylation, and facial aging phenotypic signals, we aimed to determine whether they are driven by the same causal variant. Colocalization analysis is based on five hypotheses: H0: no colocalization (i.e., the association signals of the two traits are independent); H1: the locus is associated solely with the first trait; H2: the locus is linked exclusively to the second trait; H3: both traits show an association with the locus, but they are driven by distinct causal variants (absence of colocalization); H4: the locus is associated with both traits, which share a common causal variant (colocalization is present). The posterior probabilities of these hypotheses were used to assess the likelihood of colocalization (i.e., a shared genetic signal). A higher posterior probability for hypothesis H4 suggests an increased chance of colocalization.

### 4.6. Mediation Analysis Using a Two-Step Mendelian Randomization Approach

To assess the potential mediating effects of plasma metabolites, we utilized GWAS summary data on plasma metabolites from approximately 8000 individuals of European descent. First, we evaluated the influence of the target gene expression on plasma metabolites; second, we assessed whether these metabolites further impacted disease risk. The “product of coefficients” method was employed in this analysis, which included 1400 plasma metabolites to explore the indirect association between gene expression and disease risk. Metabolites that were significant in both steps of the two-step Mendelian randomization analysis (*p* < 0.05) were identified as potential mediators.

### 4.7. Single-Cell RNA Sequence (scRNA-Seq) Analysis

The scRNA-seq data (GSE203034) of human dermal fibroblasts (ASF-4 cell line) were collected from the GEO database https://www.ncbi.nlm.nih.gov/geo/ (accessed on 28 October 2024). The ASF-4 cell line was derived from the inner side of the upper arm of a 36-year-old male donor. The dataset analyses were conducted in accordance with the methodologies described in [32]: (i) quality control: cells expressing fewer than 3 genes, genes detected in fewer than 200 cells, and cells with more than 5% mitochondrial gene percentages were filtered out; (ii) NormalizeData function was conducted to normalize the raw read counts; (iii) FindVariableGenes function was utilized to identify the 2000 highly variable genes; (iv) principal component analysis was performed based on these highly variable genes; (v) after the RunUMAP function, cells were clustered using the FindClusters function using the resolution of 0.2; and (vi) the cell types were annotated based on the expression of mark genes.

### 4.8. Statistics Analysis

All computational procedures were executed in R (version 4.3.2). Specifically, the TwoSampleMR and Mendelian Randomization R packages were used to conduct Mendelian randomization analyses. Additionally, the MR-PRESSO R package was applied to detect pleiotropy and make necessary corrections for outliers. Colocalization analysis was performed through the coloc R package. LD clumping was carried out using the plink tool. Multiple testing correction was based on the Benjamini–Hochberg method, utilizing the built-in *p*. adjust function in R. Visualization of results was conducted using the ggplot2 R package.

## Figures and Tables

**Table 1:** Genes targeted by inhaled corticosteroid medications, identified through the DrugBank and DGldb databases.

Drug Class	Drug Name	Target Gene(s)	
		DGldb	DrugBank
Inhaled Corticosteroids	Beclomethasone	NFE2L2, IL1B, NR3C1 PTH, IL2, CYP1A2 CYP2D6, AR, HIF1A ANXA1, CYP2C19, CYP2C9 SERPINA6, IL2RA, CYP3A4 CSF2, BGLAP, ZNF432	NR3C1
	Budesonide	ZNF432, EDN1, FCER2 NR3C1, CYP1A2, CYP2C9 CYP2C19, NR1I2, CYP3A4 GLCCI1, CYP2D6	NR3C1, ANXA1
	Ciclesonide	NR3C1, SERPINA6, AR	NR3C1, SERPINA6
	Fluticasone	TNFRSF8, NR3C1, CCL5 CSF2, PLA2G4A, CD163 ORMDL3, IL1B, CYP3A5 ZNF432, GLCCI1, CA10 HSPA4, IL10	NR3C1, PGR, PLA2G4A NR3C2
	Fluticasone Furoate	NR3C1	NR3C1, PGR, NR3C2
	Mometasone Furoate	CCL11, NR3C1, AR CD44, IDH1,HSPB1 NFE2L2, ANXA1	NR3C1, PGR
	Flunisolide	CYP1A2, CYP3A4, CYP2C19 NR3C1, GLCCI1, CYP2C9 TBXT, PLA2G4A, CYP2D6 AR	NR3C1

## Data Availability

Data substantiating the conclusions of this research can be obtained from the corresponding author upon a justified inquiry.

## Supplementary Materials

The following supporting information can be downloaded at https://www.mdpi.com/article/10.3390/ph18060846/s1: Table S1: Results of Mendelian randomization within the scope of inhaled corticosteroid (ICS) drug target genes identifying genes associated with facial aging risk; Table S2: Mendelian randomization analysis results of ORMDL3 gene and facial aging risk based on GTEx V8 whole-blood data; Table S3: Colocalization analysis results of posterior probabilities for inhaled corticosteroid (ICS) drug target gene ORMDL3 and facial aging; Table S4: Mendelian randomization results from ORMDL3 gene methylation to ORMDL3 gene expression in blood; Table S5: Colocalization results of posterior probabilities for ORMDL3 gene methylation and ORMDL3 gene expression; Table S6: Mendelian randomization results from ORMDL3 gene methylation to facial aging risk; Table S7: Colocalization results of posterior probabilities for ORMDL3 gene methylation and facial aging risk; Table S8: Summary of Mendelian randomization estimates for the effect of ORMDL3 gene expression on 1400 metabolites; Table S9: Summary of Mendelian randomization estimates for the effect of ORMDL3 gene expression on selected metabolites (IVW method, *p* < 0.05); Table S10: Summary of Mendelian randomization estimates for the causal effects of selected metabolites on facial aging; Table S11: Results of a two-sample Mendelian randomization study with facial aging as the exposure and 5-hydroxylysine levels as the outcome; Table S12: Results from a two-Step Mendelian randomization mediation analysis; Table S13: Details of the data sources used in this study; Table S14: Target gene names and drug actions of inhaled corticosteroids (ICS); Table S15: Genetic instruments for target genes of inhaled corticosteroid (ICS) drugs; Table S16: Genetic instruments for ORMDL3 gene based on whole-blood data from GTEx V8: Table S17: Genetic instruments for ORMDL3 gene-associated methylation.

## Abbreviations

The following abbreviations are used in this manuscript:

CI	Confidence interval
ECM	Extracellular matrix
eQTL	Expression quantitative trait loci
FDR	False discovery rate
GWAS	Genome-wide association study
ICSs	Inhaled corticosteroids
IV	Instrumental variable
IVW	Inverse variance weighted
LD	Linkage disequilibrium
mQTL	Methylation quantitative trait loci
MR	Mendelian randomization
MR-PRESSO	Mendelian Randomization Pleiotropy RESidual Sum and Outlier
OR	Odds ratio
ROS	Reactive oxygen species
scRNA-seq	Single-cell RNA sequencing
SNP	Single-nucleotide polymorphisms
